# Depression in autism spectrum disorder: a scoping review of pharmacologic, neuromodulation, and psychosocial interventions

**DOI:** 10.3389/fpsyt.2026.1906750

**Published:** 2026-10-29

**Authors:** Usman Ghumman, Celine Shafee, Lina Altarawneh, Maria Iorini, Hagar Kandel, Nikhilesh Anand, Alissa Lopez, Jacqueline Mendoza, Emma Romero, Anthony Dang, Aisha Saeed, Leonardo Magisano-Lamraoui, Tolga Atilla Ceranoglu, Ricardo Belmares, Gagan Joshi, Bharathi Gadad

**Affiliations:** 1 The Alan and Lorraine Bressler Clinical and Research Program for Autism Spectrum Disorder, Massachusetts General Hospital, Boston, MA, United States; 2 Department of Psychiatry, Harvard Medical School, Boston, MA, United States; 3 Northeastern University, Boston, MA, United States; 4 College of Medicine, Qatar University, Doha, Qatar; 5 School of Medicine, The University of Texas Rio Grande Valley (UTRGV), Harlingen, TX, United States; 6 Riphah International University, Islamabad, Pakistan; 7 Lexington High School, Lexington, MA, United States

**Keywords:** autism spectrum disorder, cognitive behavioral therapy, major depressive disorder, neuromodulation, pharmacotherapy, transcranial magnetic stimulation

## Abstract

**Introduction:**

Major depressive disorder is common in autism spectrum disorder, but diagnosis and treatment are complicated by symptom overlap, atypical presentations, alexithymia, and limited ASD-specific evidence.

**Methods:**

We conducted a scoping review of PubMed, PsycINFO, and reference lists for studies published from 1995 through 2025 examining pharmacologic, neuromodulation, and psychosocial interventions for depressive symptoms in ASD.

**Results:**

Psychosocial interventions, including autism-adapted cognitive behavioral therapy, mindfulness-based therapy, dialectical behavior therapy-informed approaches, behavioral activation, PEERS, exercise, and animal-assisted therapy, were associated with improvements in depressive symptoms in open-label studies and small randomized trials. Neuromodulation findings were preliminary, while evidence for electroconvulsive therapy and deep brain stimulation was primarily case-based. Pharmacologic recommendations were based largely on expert consensus and indirect evidence rather than ASD-specific controlled trials.

**Discussion:**

Depression treatment in ASD remains understudied. Larger controlled trials using ASD-validated depression measures, broader representation across support needs, and assessment of functional and long-term outcomes are needed.

## 1 Introduction

Autism spectrum disorder (ASD), defined by social communication differences, restricted/repetitive behaviors, and sensory processing variations, affects almost 1 in 31 children, with prevalence rising globally (1). Psychiatric comorbidities are common in ASD, with Simonoff et al. reporting that 70% of children had at least one comorbid disorder and 41% had two or more. Joshi et al. demonstrated a substantial psychiatric comorbidity burden in psychiatrically referred youth with ASD. Depressive disorders are also prevalent, with pooled lifetime estimates of 14.4% in ASD overall and 37% in adults with ASD. (2–5).

Major depressive disorder (MDD) manifests as persistent low mood, anhedonia, worthlessness, and cognitive/somatic symptoms, driving individual morbidity, relational strain, and more than $300B annual U.S. economic costs (6, 7). In individuals with ASD, social adversities amplify risk: bullying, employment barriers, social isolation, and loneliness, all recognized contributors to depression (8–11).

Yet evidence remains fragmented. Prior studies exhibit methodological heterogeneity (e.g., age, unvalidated scales, diagnostic overshadowing), likely underestimating burden. No standardized treatment pathway exists; clinicians extrapolate from neurotypical data despite sparse ASD-specific evidence across pharmacologic, psychosocial, and neuromodulation domains (5, 12).

Throughout this review, the term “individuals with autism spectrum disorder (ASD)” is used for consistency, while recognizing that both person-first and identity-first language are preferred by different members of the autism community. This PRISMA-ScR-guided scoping review synthesizes evidence from PubMed, PsycINFO, and reference list searches from 1995 to 2025 on pharmacologic, neuromodulation, and psychosocial treatments for depressive symptoms in individuals with ASD, with the goal of providing clinicians, patients, and families with a clearer overview of available options and the strength of the supporting evidence.

## 2 Materials and methods

### 2.1 Study design

We conducted a scoping review of pharmacologic, neuromodulation, and psychosocial interventions for depressive symptoms in ASD guided by the PRISMA extension for Scoping Reviews (PRISMA-ScR) framework (13). Given the limited number of ASD-specific depression intervention studies, the review also included indirect or clinically relevant evidence from ASD studies targeting related psychiatric symptoms (e.g., anxiety, emotional dysregulation, irritability) when these interventions are commonly used or recommended in the management of depression in ASD.

### 2.2 Data sources and search strategy

We searched PubMed and PsycINFO databases for studies published between December 1, 1995, and December 1, 2025. Search terms combined Medical Subject Headings (MeSH) and keywords related to autism spectrum disorder (‘autism spectrum disorder,’ ‘autism,’ ‘Asperger syndrome’), depression (‘major depressive disorder,’ ‘depression,’ ‘depressive symptoms’), and intervention-related study designs (‘clinical trial,’ ‘randomized controlled trial,’ ‘intervention’). Reference lists of included primary studies and relevant systematic reviews were manually screened to identify additional eligible primary studies. Records identified through manual reference-list screening were imported into Covidence along with records retrieved from the electronic database searches. Duplicate records were removed in Covidence prior to title and abstract screening. Only articles published in English were included. PubMed and PsycINFO were selected because they provide broad coverage of biomedical and mental health literature relevant to ASD and depression. To improve study identification, reference lists of included studies and relevant reviews were also manually screened for additional eligible articles. Because the original database-specific search strings were not retained, a PubMed search strategy was reconstructed based on the documented methodology and scope of the review and is provided in the Supplementary Material. This reconstructed strategy is intended to provide readers with a more concrete representation of the search approach and should not be interpreted as the original search string.

### 2.3 Eligibility criteria

Inclusion criteria comprised peer-reviewed articles reporting clinical outcome data in participants of any age with a confirmed ASD diagnosis (based on ADOS/ADI-R, DSM/ICD criteria, or clinical judgment). Eligible studies included interventions targeting depressive symptoms, major depressive disorder, internalizing symptoms, emotional dysregulation, suicidality, anxiety, or other psychiatric outcomes clinically relevant to depression management in ASD. Outcomes could include validated depression scales [e.g., Hamilton Depression Rating Scale (HDRS), Beck Depression Inventory-II [BDI-II], Patient Health Questionnaire–9 (PHQ-9), Children’s Depression Inventory–2 (CDI-2), Montgomery-Åsberg Depression Rating Scale (MADRS)], clinician-rated depressive symptom improvement, or related affective and internalizing outcomes.

Interventional study designs included randomized controlled trials (RCTs), quasi-experimental studies, open-label trials, pilot studies, case series, and case reports. Systematic reviews and meta-analyses were used to identify additional eligible primary studies through manual reference-list screening and to provide contextual background where appropriate but were not included as evidence units in this review. Interventions included pharmacologic treatments (e.g., antidepressants, antipsychotics, cannabinoids, and ketamine/esketamine), neuromodulation approaches (e.g., repetitive transcranial magnetic stimulation [rTMS], intermittent theta burst stimulation [iTBS], electroconvulsive therapy [ECT], deep brain stimulation [DBS], transcranial direct current stimulation [tDCS]), and non-pharmacological approaches (e.g., CBT, DBT, Mindfulness-Based Interventions, behavioral activation, social skills interventions, exercise, animal-assisted therapy, nutraceuticals, and microbiota-focused interventions.).

Given the limited number of ASD-specific intervention studies targeting major depressive disorder or depressive symptoms, we intentionally adopted broader eligibility criteria. Studies evaluating clinically related outcomes (e.g., anxiety, emotional dysregulation, irritability, suicidality, repetitive negative thinking, internalizing symptoms, and psychological well-being) were included when the intervention was considered clinically relevant to depression management in individuals with ASD. Studies that directly assessed depressive symptoms or MDD were classified as providing direct evidence, whereas studies assessing only clinically related psychiatric outcomes were classified as providing indirect evidence. Studies assessing both depression-specific and adjacent psychiatric outcomes were classified as direct evidence at the study level, with both outcome types recorded during data extraction. At the intervention level, “direct plus indirect” indicated that the evidence base included both depression-specific studies and studies assessing only related psychiatric outcomes. Classification was performed independently by C.S. and L.A. during data extraction based on the prespecified eligibility criteria. Discrepancies were resolved by consensus or, when necessary, through consultation with a third reviewer (U.G.). Throughout the review, direct evidence was interpreted separately from indirect evidence when evaluating the evidence supporting depression treatment in ASD. Exclusion criteria included studies involving mixed ASD and non-ASD populations unless results for individuals with ASD were reported separately, non-interventional or associative studies without an eligible intervention, preclinical or mechanistic studies without clinical outcomes, narrative reviews without primary or synthesized clinical data, conference abstracts without sufficient methodological detail, and studies unrelated to psychiatric or emotional outcomes relevant to depression in ASD.

### 2.4 Study selection

C.S. and L.A. independently screened titles/abstracts, then full texts using standardized forms. Discrepancies were resolved by discussion between the two primary reviewers or consultation with a third reviewer, U.G. The PRISMA-ScR flow diagram is shown in **Figure 1**.

**Figure 1:**
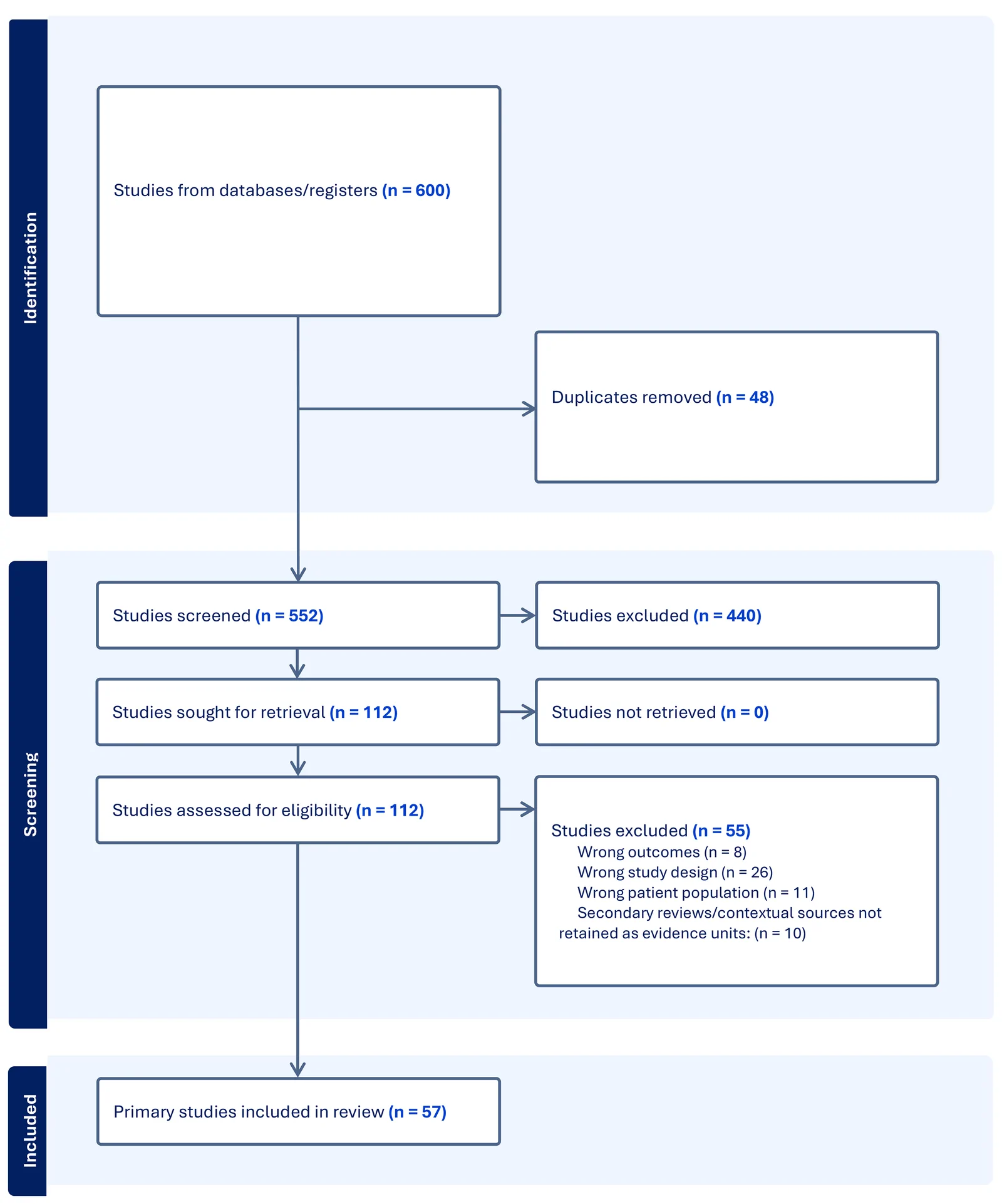
PRISMA-ScR flow diagram for identification and selection of included studies.

### 2.5 Data extraction

An individually developed data extraction form captured study characteristics, including study design (randomized controlled trials [RCTs], open-label studies, and case series); population characteristics (age, sex, ASD diagnostic method, IQ and verbal communication proficiency, and depression severity); intervention details for psychopharmacologic and neuromodulation studies (agent, protocol, dose, duration, and ASD-specific adaptations); comparator (placebo, treatment as usual, or waitlist); outcomes (primary depression scale scores, response and remission rates, adverse events, and follow-up timing); and findings (effect sizes, p-values, and confidence intervals, where reported).

### 2.6 Intervention categorization and evidence hierarchy

Interventions were categorized into three domains: non-pharmacological (psychotherapy including CBT, DBT, mindfulness; skills training such as PEERS; lifestyle and complementary interventions including exercise, animal-assisted therapy, nutraceuticals, and microbiota-focused interventions), pharmacologic (antidepressants comprising SSRIs, SNRIs, atypical antidepressants; antipsychotics; cannabinoids; ketamine/esketamine), and neuromodulation (TMS protocols including rTMS and iTBS; ECT; DBS). Evidence from included primary studies was summarized according to study design, including case reports and case series, open-label studies, observational studies, and randomized controlled trials. Systematic reviews and meta-analyses were used only for citation searching and contextual synthesis.

### 2.7 Data synthesis

Substantial heterogeneity across studies, including differences in participant age ranges, depression outcome measures, intellectual and verbal functioning, and intervention protocols, precluded quantitative meta-analysis. Therefore, findings were synthesized narratively by intervention domain. Studies were further distinguished according to whether depressive symptoms were directly measured or whether they evaluated clinically related psychiatric outcomes. Direct evidence was given greater interpretive weight when discussing treatment efficacy for depression, whereas findings derived from adjacent symptom domains were interpreted as indirect, hypothesis-generating evidence. Additional emphasis was placed on ASD-specific adaptations, methodological limitations, and major gaps within the literature.

Because this review was conducted as a scoping review, a formal risk-of-bias or certainty-of-evidence assessment was not performed. Instead, evidence was interpreted descriptively with consideration of study design, sample size, presence of comparator groups, directness of the depression outcome, duration of follow-up, and methodological limitations. The evidence-strength categories presented in **Figure 2** represent a pragmatic descriptive summary of the available literature rather than a formal certainty-of-evidence assessment or comparative ranking of treatment efficacy. Greater interpretive weight was given to larger or controlled studies directly assessing depressive symptoms or MDD, whereas smaller uncontrolled studies, case series, case reports, and studies assessing only related psychiatric outcomes were interpreted more cautiously.

**Figure 2:**
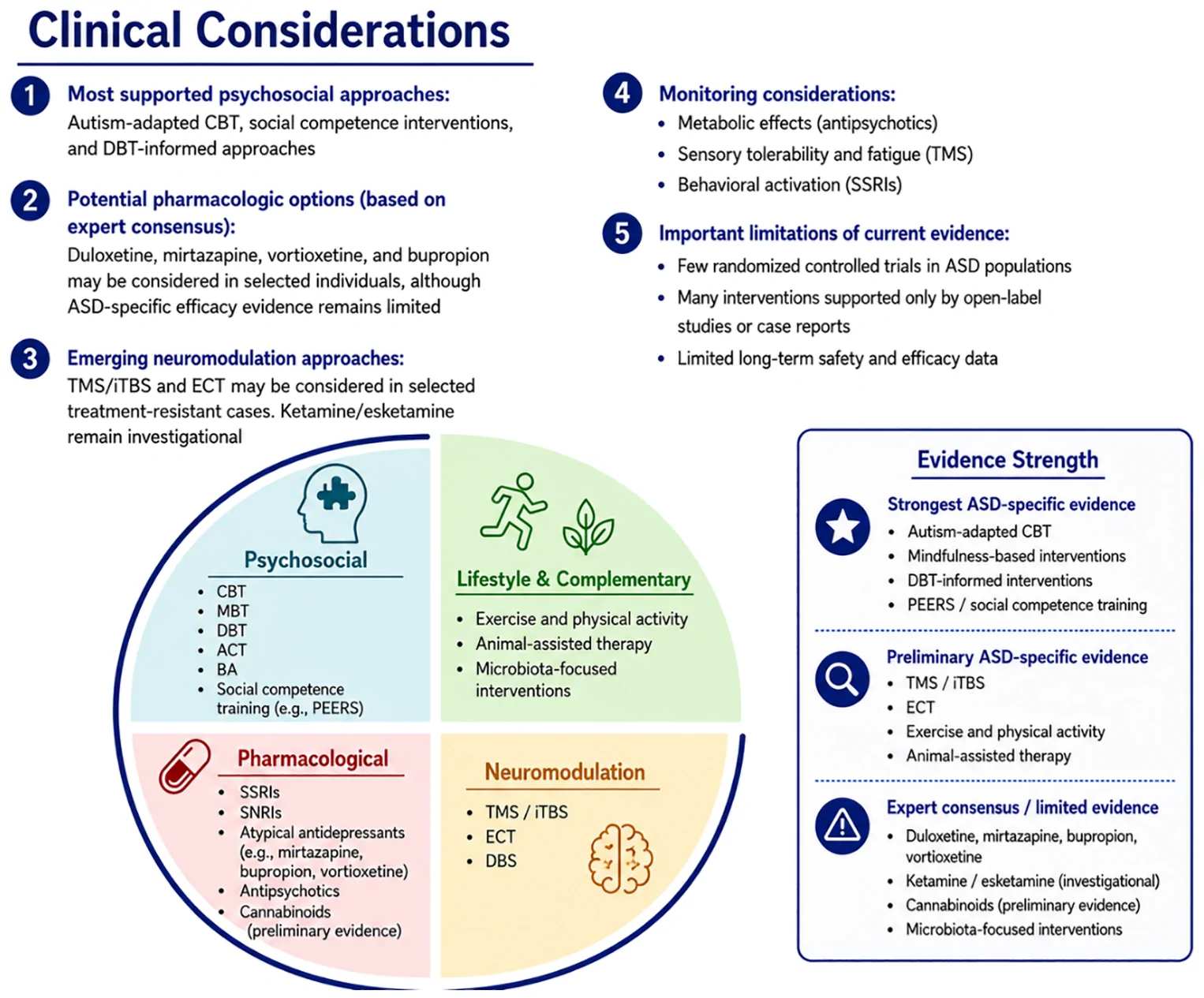
Overview of non-pharmacological, pharmacologic, and neuromodulation interventions for depression in ASD.

## 3 Results

Study selection: The search yielded 552 records after duplicate removal. Title and abstract screening excluded 440 records, and 112 full-text articles were assessed for eligibility. Of these, 55 full-text records were excluded: 8 for ineligible outcomes, 26 for ineligible study design, 11 for ineligible patient population, and 10 secondary reviews or contextual sources that were not treated as evidence units. This left 57 unique primary studies included in the review (**Figure 1**).

### 3.1 Study characteristics

The 57 included studies spanned non-pharmacological, pharmacologic, and neuromodulation interventions relevant to depression in individuals with ASD. Of these, 38 studies provided direct evidence by assessing depressive symptoms or MDD, whereas 19 provided exclusively indirect evidence through clinically related psychiatric outcomes. By intervention domain, 34 non-pharmacological studies included 27 providing direct evidence and 7 providing exclusively indirect evidence; 16 pharmacologic studies included 6 direct and 10 exclusively indirect studies; and 7 neuromodulation studies included 5 direct and 2 exclusively indirect studies. Most studies focused on adolescents and adults with verbally fluent profiles and without intellectual disability, although several pediatric and mixed-age samples were also represented. Study designs included randomized controlled trials, open-label and pilot studies, case series, and case reports, with substantial variation in intervention type, duration, and outcome measures. Depression outcomes were assessed using a wide range of validated scales, including clinician-rated and self-report instruments, which contributed to methodological heterogeneity across studies. Intervention characteristics, direct and indirect evidence, populations, adaptations, and key findings are summarized in **Table 1**.

**Table 1:** Summary of non-pharmacological, neuromodulation, and pharmacologic interventions relevant to depression in autism spectrum disorder: study characteristics, direct and indirect evidence, adaptations, and key findings.

Intervention	Study types	ASD depression-specific studies	Other ASD psychiatric/related studies	Population	Evidence for depression	Key adaptations	Key findings	Key references
CBT	Randomized controlled trials, pilot studies, group intervention studies, digital intervention trials	5 studies	No indirect ASD psychiatric/related studies identified	Adolescents and adults with ASD without intellectual disability (ID), with depression	Direct	Visual support (e.g., scales, diagrams), structured sessions, behavioral activation, focus on social/interpersonal stressors, parent/family involvement, clinician training adaptations	CBT was associated with reductions in depressive symptoms when interventions were structured, adapted to autistic cognition, and focused on social stressors, although findings from digital formats were mixed.	Russell et al. (17) 18 Gerber et al. (21) Westerberg et al. (20) Santomauro et al. (19)
MBT	RCTs, open-label studies, pilot studies, telehealth interventions	9 studies	No indirect ASD psychiatric/related studies identified	Adolescents and adults with autism; may be particularly suitable for individuals with lower verbal/emotional insight	Direct	Simplified, concrete language; removal of metaphors; structured mindfulness (breathwork, body awareness); repetitive practice; guided home exercises	Reduces depressive symptoms and rumination; increases positive affect; sustained effects with practice; comparable improvements to structured CBT in some studies; guided digital interventions effective, unguided mixed	Spek et al. (25)Kiep et al. (26)Pagni et al. (27)Conner et al. (28)Drusedau et al. (29)Frolli et al. (30)Sizoo and Kuiper (31)Stecher et al. (32)Matthews et al. (33).
DBT	Pilot trials, single-arm studies, feasibility studies (limited controlled data)	3 studies	No indirect ASD psychiatric/related studies identified	Adults with ASD without intellectual disability (ID) with emotional dysregulation and/or suicidality	Direct	Simplified manuals (reduced text), visual supports (e.g., emotion thermometers), concrete skill modeling, incorporation of focused interests, structured skills training with coaching	Significant reductions in depressive symptoms, hopelessness, emotional dysregulation, and suicidal ideation; effects sustained at follow-up in at least one study; good feasibility and acceptability	Bemmouna et al. (35)Huntjens et al. (36)Bemmouna et al. (37)
ACT	Case study	1 study	No indirect ASD psychiatric/related studies identified	Adults with ASD (very limited data; one case study in adult with MDD and ASD)	Direct	ACT matrix (visual tool), reduced reliance on abstract language, structured visual categorization of internal experiences vs. behaviors, focus on values-based actions	Case study showed reduction in depressive symptoms from severe to near subclinical levels; ACT matrix may improve accessibility for individuals with ASD; overall evidence very limited	Watanabe et al. (45)
Behavioral Activation	Pilot intervention study	1 study	No indirect ASD psychiatric/related studies identified	Youth with ASD and depressive symptoms	Direct	Tailoring activities to meaning and functional goals, incorporation of social skills practice, visual support (e.g., mood thermometers), structured routines, caregiver involvement, integration of special interest	Pilot study showed significant reduction in depressive symptoms; greater behavioral activation and social engagement associated with lower depression severity; suggests BA is feasible and promising	Menezes et al. (51)
Social competence training (e.g., PEERS)	Randomized controlled trials, controlled trials, correlational studies, group intervention studies	4 studies	3 studies	Adolescents and young adults with ASD (primarily without ID) with social difficulties and depressive symptoms	Direct + Indirect	Structured group sessions, role-playing, real-world practice (homework), parent/caregiver involvement, culturally adapted modules, focus on friendship-building and peer interaction	Improved social skills, increased peer interactions and friendship quality; reductions in depressive symptoms and interpersonal problems; benefits replicated across multiple countries	Laugeson et al. (52, 57); Schiltz et al. (58)Fatta et al. (59)Yamada et al. (60)Hillier et al. (61)Rabin et al. (62)
Exercise	Observational/survey studies, intervention trials, correlational studies	2 studies	1 studies	Children and adolescents with autism	Direct + Indirect	Structured, routine-based activities, school-based delivery, incorporation of social/group components, consistent scheduling	Higher physical activity associated with lower depressive symptoms; intervention trials show improved emotional regulation and reduced depression; group-based physical activity may enhance benefits	Tse et al. (68)Keller et al. (69)García-Villamisar et al. (70)
Nutraceuticals (probiotics)	Randomized controlled trials (behavioral outcomes)	No ASD depression-specific studies	2 studies	Children with autism; general pediatric populations	Indirect	Multi-strain formulations, microbiome-targeted interventions (no standardized ASD-specific adaptations)	RCTs show no significant improvements in core ASD symptoms vs placebo; systematic reviews suggest small behavioral benefits; no direct evidence for reducing depressive symptoms in individuals with ASD.	Liu et al. (74)Santocchi et al. (75)
Animal-assisted therapy (dog-assisted therapy)	Intervention trial, meta-analysis, qualitative study, review articles	1 study	1 study	Children and adults with autism	Direct + Indirect	Use of trained therapy dogs, nonverbal interaction, structured sessions, low verbal demand environment	Intervention trial showed reduced depressive symptoms; meta-analysis shows improvements in social, behavioral, and emotional domains; qualitative reports suggest emotional benefits; overall evidence limited.	Verheggen (80)Wijker et al. (83)Shoesmith et al. (84)
Fecal microbiota transplantation	Case report (direct evidence); secondary systematic reviews (context only)	1 case report	No indirect ASD psychiatric/related studies identified	Children and adults with ASD	Direct	Donor stool transplantation; sometimes combined with multi-strain probiotics	Secondary systematic reviews reported possible improvements in ASD-related behavioral symptoms. One case study showed partial improvement in depressive symptoms; no controlled trials evaluating depression outcomes in ASD	Hassib et al. (76)Taha et al. (77)Huang et al. (86)
TMS/rTMS/TBS	Open-label trials, protocol studies	4 studies	No indirect ASD psychiatric/related studies identified	Adolescents and adults with ASD and treatment-resistant depression (MDD/PDD)	Direct	Modified stimulation protocols (e.g., TBS, unilateral vs bilateral DLPFC targeting), high-frequency and accelerated schedules, multiple daily sessions	Open-label studies reported reductions in depressive symptoms, with response rates up to ~70% and remission rates up to ~40–67%; rapid symptom improvement was observed across several protocols; generally well tolerated with mild side effects	Blank et al. (88)Gwynette et al. (89)Elmaghraby et al. (90)Noda et al. (91)
ECT	Case study (depression); studies in ASD for catatonia	1 study	No indirect ASD psychiatric/related studies identified	Adults with ASD and severe depression and/or catatonia	Direct	Use in severe, treatment-resistant cases; applicable for individuals unable to engage in psychotherapy; standard ECT protocols under anesthesia	Case study showed improvement in depressive symptoms and functioning; the strongest existing ECT evidence in ASD relates to catatonia, while evidence specific to depressive symptoms remains limited	Bird et al. (96)Mazzone et al. (97)Park et al. (98)
DBS	Case study, case series	No ASD depression-specific studies	2 studies	Adults with ASD and comorbid OCD and/or treatment-resistant depression	Indirect	Targeted electrode placement (e.g., ventral capsule/ventral striatum, vALIC), individualized neurostimulation programming	Case study and case series showed reductions in depressive symptoms (≈45% in series); evidence extremely limited; invasive intervention reserved for severe, treatment-refractory cases	Davis et al. (102)Graat et al. (103)
SSRI	Open-label studies, observational study, case report	2 studies	2 studies	Children and adolescents with ASD (primarily); limited data in depression	Direct + Indirect	No ASD-specific pharmacologic adaptations; use often guided by comorbid anxiety/OCD; alternative agents sometimes prioritized	No RCTs for depression in ASD; mixed and nonspecific benefits in early trials; high discontinuation rates; case report suggests possible benefit in augmentation; tolerability concerns (e.g., behavioral activation)	DeLong et al. (109)Fatemi et al. (110)Argonis et al. (111)Secci et al. (112)
SNRI	Open-label retrospective report	No ASD depression-specific studies	1 study	Children with ASD (no primary depression diagnosis)	Indirect	No ASD-specific adaptations; use guided by general depression/anxiety protocols	No direct evidence for treating depression in ASD; limited retrospective data suggests nonspecific mood and behavioral improvements; clinical use based on extrapolation from general population	117
Bupropion (NDRI)	Expert guidance only	No ASD depression-specific studies	No indirect ASD psychiatric/related studies identified	N/A (no ASD-specific depression studies)	Expert consensus (no ASD evidence)	No ASD-specific adaptations; use based on general depression protocols; considered for augmentation	No ASD-specific efficacy studies were identified. Clinical use based on extrapolation from general population; potential role as augmentation; safety considerations (e.g., seizure risk)	Manter et al. (108)
Mirtazapine (NaSSA)	Randomized controlled trial (anxiety), open-label study	No ASD depression-specific studies	2 studies	Children and adolescents with autism	Indirect	No ASD-specific pharmacologic adaptations; used based on comorbid anxiety, sleep, and appetite considerations	RCT showed good tolerability and safety; open-label study suggested improvement in behavioral symptoms; no direct evidence for treating depression in ASD; proposed by expert consensus	McDougle et al. (120)Posey et al. (121)
Vortioxetine	Expert guidance only	No ASD depression-specific studies	No indirect ASD psychiatric/related studies identified	N/A (no ASD-specific depression studies)	Expert consensus (no ASD evidence)	No ASD-specific adaptations; use based on extrapolation from general MDD treatment; theoretical cognitive benefits	No ASD-specific efficacy studies were identified.	Manter et al. (108)
Antipsychotics	Randomized controlled trial; secondary systematic reviews (context only)	1 study	No indirect ASD psychiatric/related studies identified	Children and adolescents with ASD (primarily)	Direct	No ASD-specific adaptations; mainly used for irritability/aggression	Mixed evidence: one RCT showed improvement; systematic review found no consistent benefit vs placebo; not first-line for depression	Iffland et al. (127)McDougle et al. (128)
Cannabinoids (THC/CBD)	RCTs, open-label studies, retrospective observational studies; secondary systematic reviews (context only)	No ASD depression-specific studies	5 studies	Children and adolescents with autism	Indirect	Variable CBD-dominant or THC/CBD formulations with gradual titration protocols; no standardized dosing approaches	Improvements were reported in anxiety, behavioral dysregulation, social communication, repetitive behaviors, and internalizing symptoms, although depressive symptoms were rarely directly measured and findings remained inconsistent. Reported adverse effects included somnolence, appetite changes, dizziness, insomnia, anxiety, and hyperactivity.	Aran et al. (131)Bilge & Ekici (132)Hacohen et al. (133)Silva Junior et al. (134)Bar-Lev Schleider et al. (135)Guimarães et al. (136);
Ketamine/Esketamine	Case Reports	3 studies	No indirect ASD psychiatric/related studies identified	Adults with ASD with treatment-resistant depression and suicidality	Direct	Use of IV ketamine or intranasal esketamine; typically adjunctive in treatment-resistant cases	Case reports described rapid improvements in depressive symptoms and suicidality; durability of benefit was variable or incompletely reported; no controlled ASD-specific trials are available; potential safety concerns include dissociation, perceptual disturbances, hypertension, and misuse risk.	Wink et al. (142)Ozgen & Brink (143)Olivola et al. (144)

Direct evidence refers to ASD intervention studies assessing depressive symptoms or major depressive disorder. Indirect evidence refers to ASD intervention studies assessing other clinically relevant psychiatric or related outcomes. ‘Direct + indirect’ indicates that the intervention’s evidence base includes both categories. Secondary reviews and expert guidance are included for context and are not counted as primary studies. CBT, Cognitive Behavioral Therapy; MBT, Mindfulness-Based Therapy; DBT, Dialectical Behavior Therapy; ACT, Acceptance and Commitment Therapy; PEERS, Program for the Education and Enrichment of Relational Skills; TMS, Transcranial Magnetic Stimulation; rTMS, repetitive Transcranial Magnetic Stimulation; TBS, Theta Burst Stimulation; ECT, Electroconvulsive Therapy; DBS, Deep Brain Stimulation; SSRI, Selective Serotonin Reuptake Inhibitor; SNRI, Serotonin–Norepinephrine Reuptake Inhibitor; NDRI, Norepinephrine–Dopamine Reuptake Inhibitor; NaSSA, Noradrenergic and Specific Serotonergic Antidepressant; THC, Δ9-tetrahydrocannabinol; CBD, Cannabidiol.

### 3.2 Non-pharmacological interventions

Psychosocial and behavioral interventions represent the most extensively studied treatment approaches for depressive symptoms in individuals with ASD. The following sections review evidence related to psychotherapy-based, lifestyle-oriented, and complementary non-pharmacological interventions in ASD populations.

#### 3.2.1 Cognitive behavioral therapy

A total of 5 studies evaluated Cognitive Behavioral Therapy (CBT)-based interventions for depressive symptoms or related internalizing outcomes in individuals with ASD. CBT is a structured psychotherapy targeting maladaptive thinking, emotions, and behaviors underlying depressive symptoms (14). Autism-adapted CBT often uses concrete language, visual support, psychoeducation, structured sessions, and family involvement to improve accessibility and engagement (15, 16).

Adapted CBT was associated with reductions in depressive symptoms across formats. The ADEPT program showed 16-week engagement with visual routine mapping (17). Individual/group CBT guides stressor identification, mood differentiation, and rewarding activities, targeting social isolation (18–20).

Available trials primarily enrolled verbally fluent adolescents/young adults without intellectual disability, consistent with CBT’s metacognitive demands. Technology-assisted CBT showed mixed findings: visual web-based growth-mindset intervention showed improvement in depressive symptoms (21), while problem-solving digital CBT failed to improve outcomes (20). Clinician-delivered, visually structured CBT appears feasible based on the available studies.

#### 3.2.2 Mindfulness-based therapy

A total of 9 studies examined Mindfulness-Based Interventions in adolescents and adults with ASD. Mindfulness-Based Therapy (MBT) emphasizes nonjudgmental present-moment awareness to reduce rumination and depressive symptoms through acceptance (22–24). In individuals with ASD, adaptations commonly use concrete instructions, body-awareness exercises, breathing practices, and structured home practice (25).

Adapted MBT was associated with reductions in depressive symptoms, rumination, and anxiety, with increases in positive affect (25). Follow-up studies suggest benefits may be maintained with structured weekly meditation practice and stressor identification (26, 27). Protocols emphasizing bodily sensations and breath awareness were associated with improvement in repetitive negative thinking (28). One pediatric mindfulness-based intervention focused on body and emotion perception did not demonstrate significant changes in depressive symptoms, although improvements in emotional understanding were reported (29).

MBT may be suitable for individuals with alexithymia or difficulty verbalizing emotional states, and some studies suggest it may offer benefits comparable to other structured psychotherapies in verbally fluent adults with ASD (28, 30, 31). Emerging digital and telehealth-based mindfulness interventions have also shown promising reductions in depressive symptoms in adolescents with ASD (32); MINDful TIME telehealth reduced adolescent depression symptoms (33).

#### 3.2.3 Dialectical behavior therapy

Three studies evaluated Dialectical Behavior Therapy (DBT)-informed interventions targeting emotional dysregulation, suicidality, and depressive symptoms in adults with ASD. DBT integrates mindfulness-based acceptance strategies with CBT-derived change techniques, including problem-solving, skills training, and behavioral analysis, to enhance emotion regulation through individual therapy, group sessions, and coaching (34). Autism adaptations simplify manuals with diagrams/images, employ emotion thermometers for intensity rating, and incorporate participants’ circumscribed interests to boost engagement (35, 36).

Though not depression-specific, DBT may reduce depressive symptoms via improved goal orientation and valued action (37). Trials in adults with ASD and emotional dysregulation, without intellectual disability, delivered 16 skills-focused sessions with individual therapy and phone coaching, with reported improvements in depression and hopelessness (35, 37).

A 26-week comprehensive model (weekly individual psychotherapy plus skills training) was associated with sustained reductions in depressive symptoms and suicidal ideation at follow-up (36).

#### 3.2.4 Acceptance and commitment therapy

One study examined Acceptance and Commitment Therapy (ACT) for depression in an individual with ASD. ACT promotes psychological flexibility by encouraging values-consistent action despite distressing internal experiences, rather than suppression (38). Established for anxiety, health behaviors, and self-help in the general population (39–41), ASD-specific evidence for depression remains very limited. Parent-focused ACT programs have also demonstrated benefits in parent, child, and family outcomes, although these studies did not specifically establish efficacy for depression in autistic youth (42, 43).

The ACT Matrix visualizes experiences across sensory vs. mental domains and toward- vs. away-from-values behaviors, facilitating concrete mapping of barriers and goals (44).

A single case study applied the Matrix to a woman in her forties with ASD and severe MDD, characterized by rumination, irritability, social isolation, and sensory avoidance. Sixteen weekly sessions emphasized visual sorting of internal barriers versus valued actions and were associated with a reduction in depressive symptoms from severe to subclinical levels. The Matrix’s visual and concrete format may improve accessibility for some autistic individuals, although evidence remains limited to a single case report (45).

#### 3.2.5 Behavioral activation

One study evaluated Behavioral Activation (BA) targeting depressive symptoms and social engagement in youth with ASD. BA treats depression by increasing rewarding activities and reducing avoidance, targeting environments low in reinforcement through monitoring, scheduling, and social skills practice (46–48). Autism adaptations prioritize functionally meaningful activities addressing loneliness, caregiver dependence, and community isolation, common depression drivers (49, 50).

Tailoring incorporates special interests, caregiver reinforcement, predictable routines, and pre-filled tracking sheets to lessen executive demands (49, 51). A pilot for youth with ASD adapted standard BA with psychoeducation, mood thermometers, visual supports, and PEERS-like social practice (activity monitoring, friendship-building, communication skills) (51).

In the pilot study, self-reported depressive symptoms declined significantly following treatment (51).

#### 3.2.6 Social competence training

A total of 7 studies examined the Program for the Education and Enrichment of Relational Skills (PEERS) and other social competence interventions in adolescents and young adults with ASD. Four studies directly assessed depressive symptoms, whereas three provided indirect evidence through social or related outcomes. Social competence interventions aim to improve communication, empathy, and conflict resolution (52, 53). Supportive peer relationships buffer depressive symptoms in youth (54, 55) and are particularly relevant for youth with ASD, who often experience social isolation, rejection, and bullying, all risk factors for depression (50, 56).

The PEERS program uses structured training in making and keeping friends, conversational skills, handling conflict, and coping with teasing (52). Multiple trials reported improvements in social functioning and increased peer engagement (52, 57). Depressive symptoms were directly assessed in studies by Schiltz et al. (58)Fatta et al. (59)Yamada et al. (60), and Hillier et al. (61), whereas the two Laugeson studies and Rabin et al. (62) provided indirect evidence.

Successful implementations across countries show cross-cultural adaptability with consistent improvements (59, 60, 62). Programs integrating vocational skills and community activities were also associated with reductions in depressive symptoms in young adults with ASD (61).

#### 3.2.7 Lifestyle, complementary, and psychosocial interventions

Skills-based, lifestyle-oriented, and complementary interventions may be particularly beneficial for individuals with ASD who have limited emotional awareness or difficulty engaging in verbally driven therapies. The following sections review evidence related to exercise and physical activity, nutraceuticals, and animal-assisted therapy in ASD populations. These approaches aim to increase structure around positive experiences, enhance emotional regulation, and promote meaningful community participation (63). Healthy eating, regular physical activity, and inclusive social opportunities may further support mood by fostering mastery, pleasure, social connectedness, and a sense of accomplishment (63).

##### 3.2.7.1 Exercise and physical activity

Three studies evaluated exercise and physical activity interventions with depressive symptoms and related emotional outcomes in youth and adults with autism. Physical activity has been associated with improvements in mood, self-esteem, stress regulation, and overall psychological well-being (64, 65). Meta-analytic evidence in broader populations links higher levels of physical activity to lower rates of depression (66). Individuals with ASD often face barriers to regular physical activity participation (67). Structured activity programs were associated with improved emotional regulation and behavioral functioning.

Structured activity programs were associated with improved emotional regulation and behavioral functioning. A 12-week school-based jogging program was associated with emotional expression and self-awareness (68). The “Con-Tatto” program, combining walking training and social-skills sessions, was associated with reductions in depressive symptoms (69). Additional recreational exercise-based interventions were associated with improvements in personal well-being, although depression-specific effects were less consistent (70).

##### 3.2.7.2 Nutraceuticals

Two studies examined nutraceutical and microbiome-focused interventions in ASD, although none specifically targeted depression outcomes. Probiotics are live, non-pathogenic microorganisms intended to restore gut microbiota balance (71). The microbiome-gut-brain axis modulates glial function and neuroimmune signaling (72). Distinct microbiota alterations characterize both ASD and depression (73).

Clinical studies primarily examine behavioral rather than mood outcomes. Two RCTs of Lactobacillus plantarum PS128 and DSF probiotic in children with ASD found no significant differences versus placebo (74, 75). Secondary reviews have reported small to moderate behavioral improvements with probiotics, with multi-strain formulations outperforming single-strain products (76, 77).

No trials have directly evaluated probiotics for depression in youth with ASD. Broader pediatric populations show potential for certain strains and omega-3 fatty acids (78).

##### 3.2.7.3 Animal-assisted therapy

Two studies examined animal-assisted interventions in individuals with ASD, including one depression-specific trial. Animal-assisted therapy (AAT) uses trained animals, commonly dogs or horses, to support emotional well-being (79). Non-verbal animal communication may be more accessible for individuals with ASD who struggle with verbal or introspective demands (80). Companion animals support emotional development (81), and meta-analyses show AAT positively influences cognitive, social, behavioral, and emotional functioning (82).

Evidence specific to depression is limited but encouraging. A 10-week dog-assisted therapy program was associated with reductions in depressive symptoms in adults with ASD (83). Early findings suggest AAT may benefit individuals who find traditional talk-based therapies challenging (79, 80). Parental reports highlight similar benefits of dog ownership (84).

##### 3.2.7.4 Microbiota-focused interventions

One primary study examined microbiota-focused interventions in ASD, including one case report with depression-related outcomes. Fecal microbiota transplantation (FMT) modulates the gut–brain axis (85). Secondary reviews have reported possible behavioral benefits associated with FMT and probiotic interventions in ASD (76, 77). One case report showed partial improvement in depression post-FMT (86). Randomized controlled trials with standardized depression measures are needed.

### 3.3 Neuromodulation

Neuromodulation approaches are increasingly being investigated as potential interventions for severe or treatment-resistant psychiatric symptoms in individuals with ASD (87). The following sections review evidence related to transcranial magnetic stimulation (TMS), electroconvulsive therapy (ECT), and deep brain stimulation (DBS) in ASD populations.

#### 3.3.1 Transcranial magnetic stimulation

Four studies evaluated transcranial magnetic stimulation (TMS)-based interventions in individuals with ASD, including depression-specific open-label trials. TMS and theta-burst stimulation (TBS) protocols are increasingly being investigated as neuromodulation approaches for treatment-resistant depression in ASD. Four open-label trials suggest potential benefits for adolescents/adults with ASD with treatment-resistant MDD/PDD. Unilateral iTBS to left DLPFC or bilateral iTBS/cTBS (600 pulses/session, 3 sessions/day × 10 days, 90% motor threshold) was associated with rapid reductions in depressive symptoms across MDD scales (88). High-frequency rTMS to the left DLPFC (3000 pulses/session, 10 Hz, 25 sessions/6 weeks) in a small open-label sample (n = 13) reported response rates up to 70% and remission rates up to 40% (89). Accelerated TBS (10 days) was associated with a reduction in symptoms and improved cognitive flexibility (90); intensive TBS (30 sessions) was associated with Hamilton Depression Rating Scale (HDRS) remission in approximately 67% of participants (n = 18; 91).

TMS was well-tolerated despite ASD sensory hypersensitivity across 4 studies; adverse events were generally mild and transient (irritability, scalp discomfort, headache, fatigue) (88, 89, 91).

#### 3.3.2 Electroconvulsive therapy

One primary study examined electroconvulsive therapy (ECT) in an individual with ASD and provided direct evidence for depressive outcomes. ECT induces controlled seizures under anesthesia for severe, treatment-resistant disorders (92, 93). It can produce faster responses than pharmacologic or psychologic treatments for severe depression, mania, catatonia, and schizophrenia (94, 95).

A case report found that maintenance ECT was associated with remission of depressive symptoms in a 30-year-old adult with ASD, chronic major depression, and catatonia (96). Other reports of ECT in ASD have primarily focused on catatonia and are discussed as contextual evidence rather than included primary evidence units (97, 98).

#### 3.3.3 Deep brain stimulation

Two studies evaluated deep brain stimulation (DBS) in individuals with ASD and severe psychiatric comorbidity and were classified as providing indirect evidence relevant to depression. DBS involves stereotactic electrode implantation connected to an implantable pulse generator (99, 100). It has been utilized to treat movement disorders and has also been explored for aggression/self-injury (101).

ASD depression data are limited to two studies with comorbid OCD: one case and one case series. Ventral capsule/ventral striatum DBS was associated with reductions in depressive symptom scores in a 44-year-old man with ASD (102), and ventral anterior limb of the internal capsule (vALIC) DBS was associated with an approximately 45% depressive symptom reduction in 6 refractory OCD/ASD patients (103).

### 3.4 Pharmacologic treatments

Pharmacologic management of depressive symptoms in individuals with ASD remains limited by the paucity of ASD-specific depression trials. Consequently, much of the available evidence is derived from expert consensus, case reports, observational studies, or extrapolation from broader psychiatric outcomes in individuals with ASD and from treatment studies conducted in the general population. The following sections review evidence related to antidepressants, antipsychotics, cannabinoids, and ketamine/esketamine.

#### 3.4.1 Antidepressants

##### 3.4.1.1 Selective serotonin reuptake inhibitors

Four studies examined SSRI use in individuals with ASD, including two studies providing direct evidence for depressive outcomes and two providing indirect evidence. SSRIs increase synaptic serotonin by inhibiting the serotonin transporter, thereby prolonging 5-HT signaling (104–106). Although SSRIs are first-line treatments for major depressive disorder and anxiety in the general population (107), their use in ASD has primarily targeted anxiety and obsessive-compulsive symptoms rather than depression (and there are no RCTs specifically for ASD depression).

Given the limited ASD-specific efficacy data and concerns regarding tolerability in some individuals with ASD, recent expert consensus has suggested considering duloxetine, mirtazapine, bupropion, or vortioxetine in selected patients (108). However, these recommendations are based primarily on expert opinion rather than randomized controlled studies in individuals with ASD and depression. Two open-label fluoxetine studies reported nonspecific behavioral improvements at 0.2–1.4 mg/kg/day, but depressive symptoms were not clearly quantified (109, 110). Two additional studies provided direct evidence relevant to depression treatment in ASD (111, 112).

##### 3.4.1.2 Serotonin–norepinephrine reuptake inhibitors

One study examined SNRI use in individuals with ASD, although no studies specifically evaluated depression outcomes. SNRIs block the serotonin transporter (SERT) and norepinephrine transporter (NET), enhancing 5-HT/NE signaling (113–115). Established for MDD/GAD (116). Recent expert consensus has suggested duloxetine as a potential treatment option for individuals with ASD, depression, and comorbid anxiety (108); however, this recommendation is based on expert opinion and is not supported by ASD-specific controlled depression trials. One open-label study (n = 8 children with ASD, no depression diagnosis) reported qualitative mood improvements with venlafaxine (117). Duloxetine use remains off-label in ASD.

##### 3.4.1.3 Atypical antidepressants

Two studies and expert guidance sources discussed atypical antidepressants in ASD, although direct depression-specific evidence remains limited. Atypical antidepressants including mirtazapine, bupropion, and vortioxetine have been increasingly considered by expert consensus because of potential tolerability advantages over SSRIs in selected individuals with ASD.

Bupropion (NDRI): Inhibits dopamine/norepinephrine transporters (118). Bupropion is FDA-approved for MDD in the general population. Recent expert consensus has proposed it as a potential treatment or augmentation option for depression in individuals with ASD; however, no ASD-specific efficacy studies have evaluated bupropion for depression (108). Bupropion should be used cautiously in individuals with an increased risk of seizures.

Mirtazapine (NaSSA): Antagonizes α_2_-adrenergic receptors, enhancing 5-HT/NE release with sedative/appetite effects (119). One RCT involving 32 children with ASD and anxiety found mirtazapine to be safe and tolerable (120). One open-label study involving 31 participants with ASD/PDD reported improvements in mood and insomnia (121). Although recent expert consensus suggests mirtazapine as a potential treatment option for depression in ASD, direct evidence supporting its antidepressant efficacy in individuals with ASD remains limited (108). Evidence for the antidepressant efficacy of mirtazapine is derived primarily from studies of non-autistic adults with depression (122).

Vortioxetine (SERT modulator/multimodal antidepressant): Multimodal SERT inhibition plus 5-HT receptor modulation (123). Cognitive benefits in MDD (124). Recent expert consensus has suggested vortioxetine as a potential treatment option for depression in individuals with ASD. No ASD-specific studies have evaluated the safety or efficacy of vortioxetine for depression in individuals with ASD.

Overall, although atypical antidepressants have been proposed by expert consensus because of potential tolerability advantages in selected individuals with ASD, direct evidence supporting their efficacy for depression in ASD remains sparse. Additional ASD-specific randomized controlled trials are needed before firm conclusions regarding their comparative effectiveness can be drawn.

#### 3.4.2 Antipsychotics

One included primary study evaluated depression-related outcomes associated with antipsychotic medication use in individuals with ASD. A secondary systematic review was also used for contextual background. Second-generation antipsychotics target D_2_/5-HT_2_A receptors for ASD irritability/aggression (125, 126). A secondary systematic review of two studies (n = 79) reported no benefit for depressive symptoms compared with placebo (127). An RCT involving adults with autistic disorder or PDD-NOS reported reductions in clinician-rated depressive symptoms with risperidone (1–10 mg/day) (128). Risperidone is associated with metabolic and neurologic risks that require clinical monitoring (129).

#### 3.4.3 Cannabis and cannabinoids

Five included primary studies examined cannabinoid-based interventions in individuals with ASD. One secondary systematic review was used for contextual synthesis. No controlled trials specifically targeted depressive symptoms. Cannabis-derived compounds including cannabidiol (CBD) and tetrahydrocannabinol (THC) modulate endocannabinoid signaling and have been investigated for behavioral and psychiatric symptoms in ASD (130). Across randomized controlled trials, open-label studies, and observational studies, cannabinoid-based interventions were associated with improvements in behavioral dysregulation, anxiety, psychomotor agitation, social communication, and internalizing symptoms in some individuals with ASD (131–134). One open-label study reported improvement in depressive symptoms following treatment with cannabis oil containing 30% CBD and 1.5% THC (135), while another prospective real-world cohort study noted reductions in internalizing symptoms over 3 months and possible improvements in anxious and depressive symptoms (136). However, findings remain inconsistent; depressive symptoms were rarely directly measured, and most studies lacked standardized depression outcome measures. A recent systematic review similarly concluded that evidence remains preliminary and primarily focused on behavioral outcomes rather than depression-specific effects (137). Reported adverse effects included somnolence, appetite changes, dizziness, insomnia, anxiety, hyperactivity, and potential cognitive or psychosis-related concerns (134).

#### 3.4.4 Ketamine and esketamine

Three case reports examined ketamine or esketamine use in adults with ASD with treatment-resistant depression. Ketamine and esketamine are NMDA receptor antagonists that enhance glutamatergic signaling, synaptic plasticity, and downstream BDNF/mTOR activity, producing rapid reductions in depressive symptoms in treatment-resistant depression (138, 139). In the general population, IV ketamine and intranasal esketamine were associated with rapid antidepressant efficacy in randomized controlled trials (140, 141). ASD-specific evidence remains limited to three case reports with no controlled ASD-specific trials. Ketamine and esketamine were associated with rapid improvements in depressive symptoms, suicidality, and social withdrawal in adults with ASD with treatment-resistant depression (142–144). Potential adverse effects include dissociation, perceptual disturbances, hypertension, misuse risk, and reports of treatment-emergent suicidality (140, 145, 146). Although these case reports suggest potential benefits in treatment-resistant depression, the current evidence is limited to isolated reports without controlled ASD-specific studies. Accordingly, ketamine and esketamine may be regarded as promising but investigational approaches pending further ASD-specific research.

## 4 Discussion

This scoping review provides a comprehensive synthesis of the published literature evaluating pharmacologic, neuromodulation, and psychosocial interventions relevant to depressive symptoms in individuals with ASD over the past three decades. Our synthesis of the included studies reveals a central challenge in neurodevelopmental psychiatry: while MDD is highly prevalent, affecting up to 37% of individuals with ASD, the evidence base driving clinical management remains deeply fragmented, underpowered, and largely extrapolated from neurotypical data. Accordingly, conclusions regarding treatment efficacy are based primarily on direct evidence from studies evaluating depressive symptoms or major depressive disorder. In contrast, findings from studies assessing related psychiatric outcomes are interpreted as indirect evidence relevant to depression management.

Depression in ASD imposes substantial clinical burden, yet treatment evidence remains fragmented because of diagnostic challenges, heterogeneous presentations, and the limited number of ASD-specific intervention trials. A recent controlled clinical study has highlighted the bidirectional relationship between ASD and MDD, with psychiatrically referred youth with ASD demonstrating markedly elevated rates of depression, while youth with MDD also show higher than expected rates of ASD comorbidity. In a large controlled clinical study, 55% of youth with ASD had comorbid MDD, while 9% of youth with MDD met criteria for ASD, with comorbidity associated with greater psychiatric burden, emotional dysregulation, impaired social competence, and poorer global functioning (147). Compared with pharmacological approaches, psychosocial interventions currently have the broadest ASD-specific evidence base for depressive symptoms, with the largest number of autism-adapted studies and the most consistent improvements reported across trials. This interpretation reflects the greater number of ASD-specific studies rather than a formal comparative assessment of evidence quality or certainty.

Psychosocial interventions currently represent the broadest area of ASD-specific research. Autism-adapted CBT, mindfulness-based approaches, DBT-informed interventions, behavioral activation, and PEERS-based social competence training were associated with improvements in depressive symptoms or related psychiatric outcomes across studies, particularly when interventions incorporated visual support, concrete structure, caregiver involvement, and explicit social skills practice (35, 51, 52). Among these approaches, PEERS and other social competence interventions currently have some of the largest controlled evidence bases, with improvements reported in social functioning, peer engagement, and depressive symptoms (57–59). Exercise-based interventions and structured lifestyle approaches were associated with improvements in emotional regulation and reductions in depressive symptom burden, although much of the available evidence was indirect (68).

By contrast, the pharmacologic evidence base for depression in ASD remains limited. SSRIs are widely used in clinical practice and have been evaluated in randomized trials for other ASD-related symptoms; however, available studies have not established efficacy for depression in individuals with ASD. Behavioral activation remains a tolerability concern (148, 149). Recent expert guidance has proposed SNRIs and selected atypical antidepressants, including mirtazapine, bupropion, and vortioxetine, as possible alternatives in selected cases, particularly when SSRI tolerability is a concern; however, these recommendations are based largely on expert opinion, open-label studies, and extrapolation from non-ASD populations (108). Evidence for antipsychotics, cannabinoids, probiotics, and microbiota-focused interventions remains preliminary and inconsistent, with few studies directly assessing depressive outcomes (86, 127, 137).

Studies of neuromodulation approaches, particularly TMS-based protocols, reported promising preliminary findings in adolescents and adults with ASD and with treatment-resistant depression. Across open-label studies, TMS, rTMS, and theta-burst stimulation protocols were associated with rapid reductions in depressive symptoms and generally favorable tolerability despite concerns regarding sensory hypersensitivity in ASD (88–90). ECT appears particularly relevant in severe cases involving catatonia, profound functional impairment, or treatment resistance, although evidence specific to depressive symptoms in ASD remains limited primarily to case reports (96). DBS and ketamine/esketamine interventions have shown encouraging preliminary signals in refractory cases, though current evidence is restricted to small case series and individual case reports (103, 142, 144).

Several important gaps remain across the literature. Most studies focused on verbally fluent adolescents and adults without intellectual disability, leaving minimally verbal individuals and those with higher support needs significantly underrepresented. Consequently, the current evidence base is most applicable to individuals with ASD with greater verbal and cognitive abilities, particularly for psychotherapeutic interventions such as CBT, DBT, ACT, and PEERS, which rely on emotional insight, verbal communication, and cognitive flexibility. By contrast, there remains very limited evidence regarding the feasibility and effectiveness of these interventions in minimally verbal individuals, those with intellectual disability, or individuals requiring higher levels of support. Accurate assessment of depression in individuals with ASD also remains a significant challenge and likely contributes to variability across treatment studies. Emotion-regulation difficulties may influence both the presentation of depression and the accessibility of standard interventions in ASD (150). Diagnostic overshadowing may lead clinicians to attribute depressive symptoms to core features of autism, while atypical presentations, including increased irritability, behavioral rigidity, or loss of previously established adaptive functioning, may not be adequately captured by conventional diagnostic criteria or depression rating scales. Furthermore, alexithymia and communication differences can limit reliable self-reporting of emotional symptoms, and many commonly used depression measures have not been specifically validated in populations with ASD. Consequently, treatment response may be underestimated or inconsistently measured across studies, reducing comparability between interventions. Additionally, functional outcomes such as quality of life, employment, adaptive functioning, and interpersonal relationships were inconsistently reported despite their clinical importance. Heterogeneity in intervention protocols, outcome measures, and follow-up duration also limits direct comparison across studies. Future research should prioritize the development and validation of autism-sensitive depression outcome measures to improve diagnostic accuracy and strengthen the evidence base for depression treatment in ASD.

Overall, current evidence suggests that depression in ASD is both common and clinically impairing yet remains understudied relative to its prevalence and functional burden. Autism-adapted psychosocial interventions currently have the broadest ASD-specific evidence base, while pharmacologic approaches remain largely guided by extrapolation from non-ASD populations and expert consensus. Neuromodulation strategies, particularly TMS-based interventions, represent an emerging area of interest with encouraging preliminary findings for treatment-resistant depression in ASD. Future research should prioritize larger controlled trials, ASD-validated depression measures, inclusion of individuals across support levels, and long-term functional outcomes to improve evidence-based treatment recommendations for depressive symptoms in ASD.

## 5 Implications for practice and future directions

### 5.1 Psychosocial interventions

Current evidence most consistently suggests potential benefit from autism-adapted psychosocial interventions, particularly CBT-, mindfulness-, DBT-, and PEERS-informed approaches, which were associated with the most consistent ASD-specific improvements in depressive symptoms across studies. Structured exercise programs, social engagement interventions, and other lifestyle-oriented approaches may be considered as useful adjunctive strategies, particularly for individuals who have difficulty engaging in verbally intensive therapies. Future research should prioritize larger controlled trials comparing different psychotherapy modalities, evaluation of long-term outcomes, and identification of patient characteristics associated with treatment response. Across psychosocial interventions, several autism-specific adaptations were commonly incorporated in studies reporting acceptable feasibility and clinical improvement regardless of the underlying therapeutic model. These included the use of visual supports, concrete and literal language, highly structured session formats, predictable routines, incorporation of circumscribed interests to enhance engagement, caregiver involvement when developmentally appropriate, behavioral rehearsal, and individualized pacing based on cognitive and communication abilities. Although these adaptations varied somewhat across psychosocial interventions (CBT, mindfulness-based interventions, DBT, behavioral activation, Acceptance and Commitment Therapy, and social competence training), their shared emphasis on reducing cognitive demands and increasing treatment accessibility likely contributed to improved engagement and reductions in depressive symptoms across studies.

### 5.2 Pharmacologic interventions

Pharmacologic management of depression in ASD remains largely extrapolated from non-ASD populations and expert consensus because of the limited number of ASD-specific medication trials. Recent expert consensus has proposed that duloxetine and mirtazapine may be considered in selected cases, particularly when tolerability concerns limit SSRI use. However, these recommendations are based primarily on expert consensus rather than ASD-specific controlled trials. Clinicians should carefully monitor behavioral activation, irritability, sleep disturbance, and suicidality when initiating antidepressants, especially SSRIs. Given the high rates of psychiatric comorbidity in ASD populations, future studies are needed to better characterize medication effectiveness, tolerability, and predictors of response in individuals with ASD and depressive symptoms.

### 5.3 Neuromodulation and emerging interventions

Neuromodulation approaches, particularly unilateral DLPFC iTBS and ECT, have been investigated in severe, treatment-resistant, or highly impairing presentations in ASD, but current evidence remains preliminary and is based largely on open-label studies, case reports, and small samples. When these interventions are used clinically, clinicians should consider sensory tolerability, cognitive effects, and the need for individualized protocol adaptations when using TMS- or ECT-based interventions in individuals with ASD (**Figure 2**).

## 6 Limitations

An important limitation of this review is the deliberate inclusion of studies evaluating psychiatric outcomes adjacent to depression, including anxiety, emotional dysregulation, irritability, suicidality, and repetitive negative thinking. This broader approach was necessary because ASD-specific intervention studies directly targeting major depressive disorder or depressive symptoms remain scarce; however, it also increased the clinical and methodological heterogeneity of the synthesized evidence. Consequently, improvements observed in these related symptom domains should not be interpreted as equivalent to improvements in depression. Throughout this review, findings derived from adjacent psychiatric outcomes were considered indirect evidence and should be viewed as hypothesis-generating rather than definitive evidence supporting antidepressant efficacy in individuals with ASD. Future research should prioritize well-designed studies using validated depression-specific outcome measures to better distinguish treatment effects on depression from improvements in overlapping psychiatric symptoms commonly observed in ASD. Additional limitations should also be considered. First, the available literature is dominated by methodologically limited evidence, including open-label trials, pilot studies, feasibility studies, case series, and case reports, with relatively few randomized controlled trials specifically targeting depressive symptoms in individuals with ASD. Second, substantial heterogeneity across studies, including differences in participant age, intellectual and verbal functioning, intervention protocols, outcome measures, follow-up duration, and depression assessment tools, precluded quantitative meta-analysis and limited direct comparison across interventions. Third, many studies relied on depression scales that have not been specifically validated in ASD populations, raising concerns regarding diagnostic overlap, atypical symptom presentation, and diagnostic overshadowing. Fourth, most included studies focused on verbally fluent adolescents and adults without intellectual disability, limiting generalizability to minimally verbal individuals and those with higher support needs. In addition, the literature search was limited to PubMed and PsycINFO and was not prospectively registered. Because the literature search was conducted iteratively during study development, the original database-specific search strings were not retained. Consequently, although the search methodology is described in detail, the exact electronic search strategies cannot be fully reproduced. Although manual reference list searches were performed to identify additional studies, relevant publications indexed exclusively in other databases (e.g., Embase, Scopus, or CENTRAL) may have been missing. Future scoping or systematic reviews should consider broader database coverage and prospective protocol registration to further strengthen methodological rigor. Finally, publication bias and selective reporting may have favored positive findings, particularly within small open-label and case-based studies. Larger controlled trials using ASD-validated depression measures and longer-term functional outcomes are needed to strengthen the evidence base for depression treatment in individuals with ASD.

## 7 Conclusion

The available evidence suggests a need for individualized, autism-adapted approaches rather than uncritical reliance on treatment extrapolation from non-ASD populations. Among the interventions reviewed, autism-adapted psychosocial approaches, particularly CBT-, mindfulness-, DBT-, and PEERS-informed approaches, were associated with the most consistently reported ASD-specific improvements in depressive symptoms or related psychiatric outcomes. Neuromodulation approaches such as iTBS and TMS remain investigational options for treatment-resistant cases; however, the overall evidence base remains limited, highlighting the need for larger ASD-specific depression trials and validated assessment frameworks across the autism spectrum. Future research should prioritize adequately powered randomized controlled trials, standardized depression outcome measures validated for ASD populations, inclusion of individuals across support levels, and evaluation of individualized multimodal treatment approaches.

## Data Availability

The original contributions presented in the study are included in the article/**Supplementary Material**. Further inquiries can be directed to the corresponding author.
