# Supplementary material for: Depression in autism spectrum disorder: a scoping review of pharmacologic, neuromodulation, and psychosocial interventions
Source: Front Psychiatry. 2026 Oct 29;17:1906750. doi: 10.3389/fpsyt.2026.1906750 (PMC13755279; doi:10.3389/fpsyt.2026.1906750)
Supplement: Supplementary file 1 [file Table1.docx]

**Supplementary Table S1. Reconstructed PubMed Search Strategy**

**Database:** PubMed

**Date range:** December 1995–December 2025

The following search strategy was reconstructed based on the documented methodology and scope of the scoping review. It is presented as a reconstructed search strategy and should not be interpreted as the original database-specific search string.

```
(
  "Autism Spectrum Disorder"[Mesh]
  OR "Autistic Disorder"[Mesh]
  OR autism*[tiab]
  OR "autism spectrum disorder*"[tiab]
  OR autistic[tiab]
  OR Asperger*[tiab]
)
AND
(
  "Depressive Disorder"[Mesh]
  OR "Depression"[Mesh]
  OR depress*[tiab]
  OR "major depressive disorder"[tiab]
  OR "depressive symptom*"[tiab]
  OR "depressive disorder*"[tiab]
)
AND
(
  "Antidepressive Agents"[Mesh]
  OR antidepressant*[tiab]
  OR SSRI*[tiab]
  OR "selective serotonin reuptake inhibitor*"[tiab]
  OR SNRI*[tiab]
  OR "serotonin norepinephrine reuptake inhibitor*"[tiab]
  OR fluoxetine[tiab]
  OR sertraline[tiab]
  OR escitalopram[tiab]
  OR citalopram[tiab]
  OR venlafaxine[tiab]
  OR duloxetine[tiab]
  OR bupropion[tiab]
  OR mirtazapine[tiab]
  OR vortioxetine[tiab]

  OR antipsychotic*[tiab]
  OR aripiprazole[tiab]
  OR risperidone[tiab]
  OR ketamine[tiab]
  OR esketamine[tiab]

  OR nutraceutical*[tiab]
  OR probiotic*[tiab]
  OR prebiotic*[tiab]
  OR microbiome[tiab]
  OR cannabis[tiab]
  OR cannabinoid*[tiab]

  OR psychotherapy[tiab]
  OR "cognitive behavioral therapy"[tiab]
  OR CBT[tiab]
  OR "behavioral activation"[tiab]
  OR "acceptance and commitment therapy"[tiab]
  OR ACT[tiab]
  OR "dialectical behavior therapy"[tiab]
  OR DBT[tiab]
  OR "mentalization based therapy"[tiab]
  OR MBT[tiab]
  OR "psychosocial intervention*"[tiab]
  OR "behavioral intervention*"[tiab]
  OR "psychological intervention*"[tiab]
  OR mindfulness[tiab]
  OR exercise[tiab]
  OR "physical activity"[tiab]
  OR lifestyle[tiab]

  OR "Animal Assisted Therapy"[Mesh]
  OR "animal-assisted therap*"[tiab]
  OR "animal assisted therap*"[tiab]
  OR "animal-assisted intervention*"[tiab]
  OR "animal assisted intervention*"[tiab]
  OR "equine-assisted therap*"[tiab]
  OR "equine assisted therap*"[tiab]
  OR "horse-assisted therap*"[tiab]
  OR "horse assisted therap*"[tiab]
  OR "dog-assisted therap*"[tiab]
  OR "dog assisted therap*"[tiab]
  OR "canine-assisted therap*"[tiab]
  OR "canine assisted therap*"[tiab]
  OR "pet therapy"[tiab]

  OR "transcranial magnetic stimulation"[Mesh]
  OR "transcranial magnetic stimulation"[tiab]
  OR TMS[tiab]
  OR rTMS[tiab]
  OR "deep TMS"[tiab]
  OR SAINT[tiab]
  OR "intermittent theta burst"[tiab]
  OR iTBS[tiab]
  OR "electroconvulsive therapy"[tiab]
  OR ECT[tiab]
)
AND
("1995/12/01"[Date - Publication] : "2025/12/01"[Date - Publication])
```

**Note.** This strategy was reconstructed retrospectively from the documented review methodology and scope to improve transparency and reproducibility; it is not presented as the original PubMed search string.
